# The Emergence of Autism Symptoms Prior to 18 Months of Age: A Systematic Literature Review

**DOI:** 10.1007/s10803-020-04618-w

**Published:** 2020-07-30

**Authors:** Amy Tanner, Katerina Dounavi

**Affiliations:** 1 Queen’s University of Belfast, Belfast, Northern Ireland; 2 School of Social Sciences, Education & Social Work, 69-71 University Street, Belfast, BT7 1HL Northern Ireland

**Keywords:** Autism spectrum disorders, Early behavioral symptoms, Systematic literature review, Early screening

## Abstract

Pre-diagnostic intervention for autism spectrum disorder (ASD) allows symptoms to be addressed as they emerge, often between six to 18 months, rather than after the full onset of the disorder. A systematic literature review, spanning the previous six years was conducted in order to provide an updated review looking at the earliest behavior symptoms of ASD. All included studies used a prospective experimental design, reported on symptoms that emerged before 18-months of age, exclusively in children who would later receive a diagnosis, and were assessed for quality. This review is the first to address this research question through the use of a systematic research design and extends the literature by following up on recommendations for future research from previous findings.

**Electronic supplementary material:**

The online version of this article (10.1007/s10803-020-04618-w) contains supplementary material, which is available to authorized users.

Various ASD signs and symptoms begin to emerge in the first year of life and can be detected between 6 and 18 months of age, however the average age of diagnosis is 4 years of age or older across North America (Centre for Disease Control 2019). Improving the identification of very early Autism Spectrum Disorder (ASD) symptoms is a priority amongst autism researchers, as earlier identification allows for earlier intervention, which is in turn key for maximizing a child’s potential and achieving optimal outcomes. More in detail, optimal outcomes in children with ASD are linked to the age at which intervention begins, with the most significant gains being observed in children who begin behavioral intervention prior to 2 years of age (Ben-Itzchak & Zachor 2007; Landa 2018; MacDonald et al. 2014).

Research examining the early signs of ASD can be categorized into three domains: (a) retrospective studies, often taken from parents’ recall of their child’s behaviors during infancy; (b) videotape reviews, consisting in home video reviews of infant and toddler behaviors from children who later on were diagnosed with ASD; and (c) prospective studies, often following a high-risk cohort from birth and documenting the emergence of symptoms as they unfold. Retrospective research, often in the form of surveys and questionnaires which probe questions such as “when did you first have concerns of your child’s development and what were they”, can be useful in identifying what stands out to parents after time passes or what symptoms may have been most salient and memorable. However retrospective research are prone to many memory errors and biases. Videotape review was an invaluable step in establishing early signs research. A landmark study by Osterling and Dawson (1994) coded home videotapes of first year birthday parties of both typically developing children and children who would later be diagnosed with autism and found that 91% of the children who later received a diagnosis engaged in significantly fewer social communicative behaviors, such as responding to their name, using gestures and looking at others. Using videotape review to collect data and observe how symptoms presented at an earlier point in time eliminates the memory errors and biases attributed to retrospective research. However, videotape reviews are prone to selection bias, in that, parents are more likely to record special moments and adaptive behavior and may be more likely to stop recording when challenging behavior emerges (Osterling & Dawson, 1994). Prospective research often follows a cohort of a specific population and records data during frequent intervals in real time, allowing for a more accurate understanding of the timing and topographies of the earliest ASD symptoms. Prospective research often relies on the use of a high risk (HR) sample, referring to a heightened genetic risk often confirmed by an older sibling with a confirmed diagnosis of ASD. A HR sample ensures a higher percentage of future confirmed cases of ASD, approximately 20% (Ozonoff et al. 2011), compared to less than 2% found in a general population or low risk (LR) sample. Group membership is further classified based on diagnostic outcomes to include HR-no ASD, HR-ASD, LR-no ASD and LR-ASD (confirmed ASD diagnosis with no known genetic risk). HR-no ASD membership may be further divided into HR-ATP (atypical development but no ASD) and HR-TD (typical development despite genetic risk). Studies may use any combination of these groups depending on the diagnostic outcome of individual participants at follow-up and the dependent variables measured. Studies have reliably demonstrated that infant to toddler developmental trajectories of HR-no ASD are often differentiated from LR-no ASD with regards to characteristics such as anticipatory responses (Landa et al. 2016; Northup et al. 2017), object exploration and fine motor skills (Ekberg et al. 2016; Kaur et al. 2015; Koterba et al. 2014; Leonard et al. 2015; Libertus et al. 2014), sharing objects (Srinvasan & Bhat 2016), eye contact and gaze (Bedford et al. 2012; Dundas et al. 2012; Gliga et al. 2015) and social engagement (Chawarska et al. 2016; Jones et al. 2015). Following the same logic, early signs in a HR-ASD population should not be assumed to generalize to a LR-ASD population and prospective research should continue to include diverse LR samples (Zwaigenbaum et al. 2015).

Early intervention is essential for children to maximize their developmental trajectory and reach full potential (Landa et al. 2018; Reinchow et al. 2012) however one of the biggest barriers to early intervention is early detection. In order to improve early detection, research must focus on the earliest manifestations of the disorder in infancy. A comprehensive literature review was conducted by Zwaigenbaum et al. (2015) summarizing key findings of early signs from research up until the end of 2013. A few notable limitations to this study is that it did not use a systematic review methodology, nor did it focus solely at early signs that differentiate the high-risk atypical or typical development from high-risk-ASD development. Although this literature review was not systematic, 419 articles were reviewed by an expert panel of 22 researchers who are highly specialized in the area of early autism symptoms, thus allowing for a wider scope of articles to be included with some discretion in order to include the most relevant articles (Zwaigenbaum et al. 2015). Six years has passed since its publication and the area of early behavior symptoms has continued to spark interest, leading to dozens more publications on the topic. As the previous literature was extremely comprehensive and included 24 articles, the current literature review focuses on the most recent findings rather than including redundant findings which have already been covered extensively by its predecessor.

It is well documented across high-risk research that the younger siblings of children with autism that do not go on to receive a diagnosis of ASD often still show developmental differences compared to low-risk no ASD children. More specifically, children who are HR-no ASD will often present with a range of less severe symptoms than HR-ASD but present with more symptoms than LR-no ASD. An important contribution of the current systematic literature review focuses on where and when children with HR-ASD differ from all other comparison groups, thereby providing a summary of early signs that are exclusive to a group that will eventually be diagnosed with ASD. This distinction was intentionally examined in order to more clearly show symptoms associated with a future ASD diagnosis rather than symptoms that are solely associated with HR group membership.

The current study aims to extend the aforementioned review by examining the subsequent 6 years and adding to our understanding of early identification of ASD by using a systematic approach and limiting studies exclusively to prospective designs that examined symptoms which were significantly differentiated before 18 months of age in a HR-ASD sample. To date, no review has examined the early symptoms of ASD which differentiate HR infant siblings who do go on to receive a confirmed diagnosis to HR infants who do not go on to receive a diagnosis (i.e. HR-ASD symptoms versus HR-ATP or HR-TD. Although a substantial amount of literature has examined early symptoms often observed in a HR sample, this review will extend the literature by examining which symptoms are exclusive to the HR-ASD group.

## Method

### Systematic Search Procedures

Systematic searches were conducted in four electronic databases: Medline, PubMed, ERIC and PsycINFO. Searches were limited to peer-reviewed journal articles, written in English and published in the previous 5 years (January 2014-Dec 2018). Identical search terms were used across all four databases which included: “early signs” or “early detection” or “early symptoms” and “prospective” and “autism” or “Autism Spectrum Disorder” or “ASD”.

**Fig. 1:**
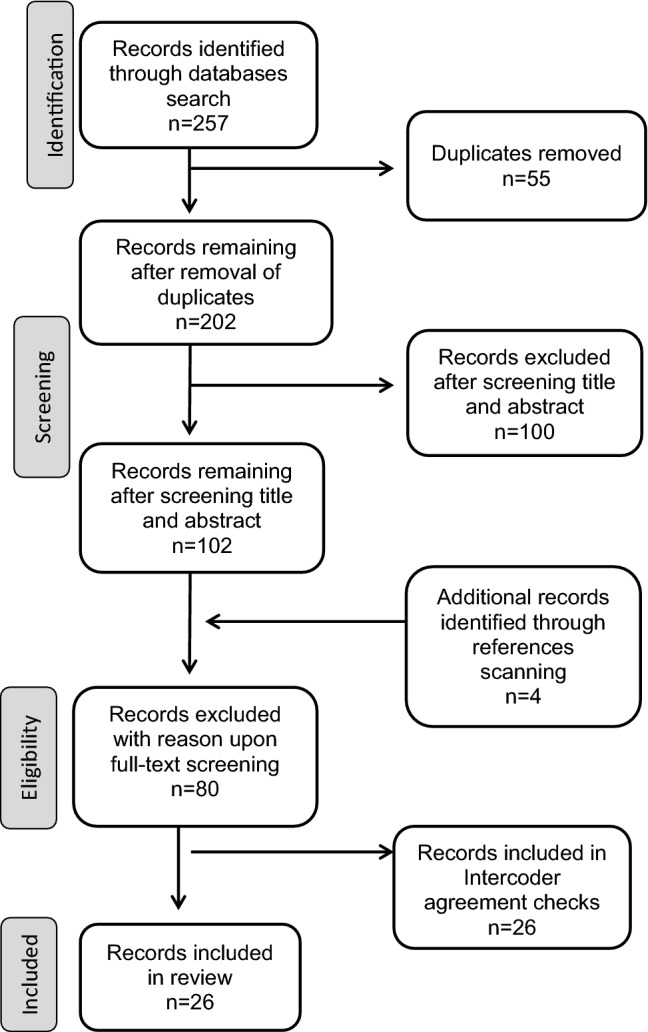
Flow-chart illustrating inclusion process

The search results generated 146 titles with abstracts, from which 85 were removed due to lack of relevance to the research question or duplicates across databases, leaving 61 relevant articles for subsequent full-text screening. Inclusion and exclusion criteria were used for screening full-texts after which 18 articles were deemed eligible for inclusion in this review (Fig. 1; Moher et al. 2010). Hand searches of the references of the 18 eligible studies were conducted resulting in the identification of an additional article deemed relevant by two independent coders and bringing the total number of eligible studies to 19. To ensure the final manuscript included the most recent literature, the search was updated by using the same methodological procedure to include articles published up until May 31, 2020. This addition resulted in 111 additional references for screening, yielding 7 additional titles included in the present review. Tables and texts references were updated to reflect the total amount of references at each stage of the screening process.

### Inclusion and Exclusion Criteria

To be included in this review, studies should meet the following criteria: (1) participants were 18 months or younger at baseline; (2) a post-screening diagnostic tool, coding system or clinical best estimate was administered to determine the presence or absence of an ASD diagnosis; (3) the symptoms were behavioral in nature and measurable during direct observation (including video recording, i.e. could be observed by a parent or during routine clinical practise); (4) symptom(s) emerged before 18 months of age in the ASD group and were differentiated from comparison groups before 18 months; (5) studies were prospective in nature; and (6) no co-occurrence of additional diagnoses was reported.

Studies were excluded if: (1) additional diagnoses were present; (2) the study did not confirm an outcome status of autism or ASD (e.g. only reported an at-risk status); (3) the symptoms required additional technology to measure, such as but not limited to eye-tracking devices, neuro-imaging, visual or audio analysis software; (4) symptoms were not significantly differentiated from comparison groups (i.e. HR-No ASD and LR) by 18 months of age.

### Data Coding and Inter-Coding Agreement

The first author conducted the initial search, removal of duplicates and removal of articles by scanning abstract and title. After these first three steps were conducted, 102 articles remained to undergo full-text screening. The first author screened 100% of the articles for inclusion and three independent coders screened 50% (51 articles) for inclusion. Of the 102 articles which underwent full-text screening, 26 articles met full inclusion criteria, with 100% agreement between the first author and independent coders. All 19 articles were coded by the first author (Table 1) and three independent coders. Articles were coded as follows: (1) participants age at baseline; (2) post-screening diagnostic tool, coding system or clinical best estimate administered to determine the presence or absence of an ASD diagnosis; (3) behavioral and measurable symptoms; (4) symptom(s) that emerged before 18 months of age in the ASD group and how these differentiated from comparison groups; and (5) quality of each study assessed using a modified version of the Observational Cohort and Cross-Sectional Studies (NIH National Heart, Lung and Blood Institute, 2017) with a quality score reported. The full-text of all 26 articles selected for inclusion was screened against inclusion and exclusion criteria and scored for quality by the primary researcher and three independent coders.

**Table 1:** Summary of Included Studies

Study	Findings	Sample	Outcome Diagnosis	Details	Quality Rating
Bedford et al. 2016	At 14 months, gaze following task and deficits in disengagement were effective markers in boys	104 Infants 5–7 months at intake 54 h 50 LR 40% male	At 36 months obtained scores over the cutoff for ASD on the ADOS-G	Infants part of larger British Autism Study of Infants AOSI severity scores used for gaze and disengagement of attention	Mod (9)
Bresnahan et al. 2015	Mothers of children with ASD more likely to report 1 or more GI symptom prospectively at 18mo (constipation, diarrhea or food intolerance) By 36 months, children with ASD had twofold increased odds of reporting GI symptoms compared to TD	45,126 Infants 6–18 months 195 ASD 4636 DD 40,295 TD	At 36 months obtained cutoff scores for ASD on the ADOS and ADI-R	Infants part of Norwegian Mother and Child Cohort Study The study uses a LR sample	High (10)
Choi et al. 2018	HR-ASD showed slower growth in fine motor skills between 6 and 24 months compared to HR-no ASD	170 Infants 71 h – no ASD 30HR-ASD 69 LR	At 36 months obtained cutoff scores for ASD on the ADOS and ADI-R	MSEL to assess fine motor	High (11)
Del Rosario et al. 2014	HR-ASD differed in 2 of the 9 temperament measures: decreased adaptability and approach	54 Infants 16 h- ASD 11 h-no ASD 27 LR	At 36 months obtained cutoff scores for ASD on the ADOS and ADI-R	Carey Temperament Scales and MSEL to assess temperament, cognition and motor abilities Data collected at 5 pts in time Infants recruited from 3 different locations	High (10)
Elison et al. 2014	HR-ASD group showed more stereotyped motor mannerisms at 12 months than HR- no ASD HR-ASD and HR-no ASD did not differ with repetitive object manipulation (both groups engaged in more than LR group)	158 Infants 30 h-ASD 75 h – no ASD 53 LR	At 24 or 36 months met DSM criteria and obtained scores over the cutoff for ASD on the ADOS	Infants part of larger Infant Brain Imaging Study Communication and Symbolic Behavior Scales- Developmental Profile (CSBS-DP) and the Repetitive and Stereotyped Movement Scales (RSMS)	High (10)
Estes et al. 2015	At 12 months HR-ASD showed less developed gross motors,	308 Infants 49 h-ASD 161 h- no ASD 98 LR- no ASD	At 24 months cutoff scores from the ADOS and ADI-R were used along with clinical best estimate to determine a diagnosis	The MSEL, VABS-II, AOSI were conducted at 6, 12, and 24 month visits	High (10)
Filliter et al. 2015	HR-ASD had lower rates of smiling than HR-no ASD	66 Infants 22 h ASD 22 h no ASD 22 LR	At 36 months obtained cutoff scores for ASD on the ADOS and ADI-R	Infants recruited from larger Canadian Infant Sibling study AOSI scores of visual tracking and disengagement of attention to assess affect	High (10)
Gammer et al. 2015	HR-ASD group showed differences in visual tracking and social referencing at 7 months and differences in orientation to name and engagement of attention at 14 months	104 Infants 17 h-ASD 36 h- no ASD -1 h (attrition) 50 LR	At 36 obtained cutoff scores for ASD on the ADOS -G and ADI-R	Infants part of larger British Autism Study of Siblings AOSI used to measure autism symptoms	High (10)
Gangi, Ibañez et al. 2014	HR-ASD showed deficits in initiating joint attention without smiling and lower levels of anticipatory smiling than HR-no ASD	82 Infants 12 h -ASD 44 h-no ASD 26 LR	At 36 months obtained cutoff scores for ASD on the ADOS	Infants part of larger Sibling Studies Measuring Infant Learning and Emotion Project Early Social Communication Scales (ESCS) to assess JA MSEL to assess expressive and receptive language	High (12)
Gangi, Schwichtenberg et al. 2018	HR-no ASD and LR risk showed increased social gaze with parent over examiner HR-ASD did not differ between context and showed overall lower rates	136 Infants 17 h-ASD 66 h -no ASD 53 LR	At 36 months met DSM criteria and obtained scores over the cutoff for ASD on the ADOS	Infants part of larger longitudinal study MSEL to assess cognitive functioning	High (10)
Gordon & Watson 2015	HR-ASD used half as many gestures as HR-no ASD between 13–15 months Gestures at 13–15 months significantly correlated with expressive and receptive language at 24 months	42 Infants 14 h- ASD 15 h-BAP (no ASD) 13 h -TD	At 24 months met cutoff on ADOS	Infants part of larger intervention study MSEL to assess cognition and language	Mod (8)
Heymann et al. 2018	HR-ASD engaged in fewer joint attention behaviors and less frequent and advanced vocalisations (primarily vowel only vocalisations)compared to HR-no ASD and HR-LD	50 Infants 9 h-ASD 15 h- LD 26 h-ND	At 36 months met DSM-IV criteria, cutoff scores on ADOS and clinical best estimate	Recruited through a university Autism Research Program and through flyers, professional referrals and word of mouth. All participants were HR and no LR sample was recruited	High (11)
Lazenby et al. 2016	HR-ASD showed reduced quantity of single-word understanding at 12 months compared to HR-no ASD	346 Infants 43 h-ASD 170 h- no ASD 133 LR	At 36 months diagnosis made by Clinical Best Estimate	Infants part of larger Baby Sibling Research Consortium MacArthur-Bates Communicative Developmental Inventories (CDI) Words and Gestures to assess communication abilities MSEL to assess language	Mod (9)
LeBarton & Landa, 2019	Deficits in visual motor integration (goal directed reach) gross motor pull to sit task and grasping at 6 months predicted ASD group membership	140 Infants 20 h-ASD 69 h- no ASD 51 LR	At 24 or 36 months DSM-IV criteria and obtained scores over the cutoff for ASD on the ADOS	Recruited through various methods: flyers, social media, community events, local providers Peabody Developmental Motor Scales-2 (PDMS-2) to assess motor skills MSEL to assess general development	High (10)
Macdonald et al. 2020	At 6 to 36 months, HR-ASD had lower cognitive abilities and at 12 months HR-ASD had lower adaptive abilities compared to HR-no ASD, however only in single-incident families	435 Infants Single-Incident Families 57 h-ASD 298 h- no ASD Multiplex Incident Families 29 h-ASD 51 h- no ASD	At 36 months cutoff scores for ADOS and clinical best estimate were used to determine ASD diagnoses	MSEL and VABS-II used for cognitive and adaptive functioning measures Developmental trajectories of HR with one older sibling with ASD (single-incident) vs. two or more older siblings with ASD (multiplex incident). HR infants from multiplex families were more than 2 × as likely to receive ASD diagnosis by 36 months than HR from single incident homes	Mod (9)
Miller et al. 2017	At 9 months of age, HR-ASD we more likely to fail to orient to their name compared to HR- no ASD and LR- no ASD	156 Infants 20 h- ASD 76 h – no ASD 60 LR – no ASD	At 36 months DSM-IV criteria and cutoff scores for ADOS determined diagnosis	AOSI press for “orients to name” was used to assess orienting to name and ADOS, MSEL & VABS-II were used to assess cognitive and adaptive functioning	High (11)
Nichols et al. 2014	HR-ASD showed lower rates of eye contact and non-social smiling compared to HR-no ASD Eye contact may serve as protective factor for HR-no ASD group	67 Infants 15 h-ASD 27 h- no ASD 25 LR	At 23 months, ADOS and DSM used to diagnose ASD or Broad Autism Phenotype	Infants part of a larger sample of longitudinal study examining early development of ASD MSEL to assess overall functioning STAT to assess social smiling	High (11)
Ozonoff et al. 2018	Declining trajectories/regression observed in 88% of HR-ASD at 12 months by using prospective dimensional recording of current functioning	230 Infants 30 h-ASD 117 h- no ASD 2 LR-ASD 81 LR no ASD	At 36 months met DSM-IV criteria and obtained scores over the cutoff for ASD on the ADOS	Does not specify how recruitment was conducted Early Development Questionnaire (EDQ) part 1 and 2 to assess current performance and lost skills	Mod (9)
Parladé & Iverson 2015	Slower acquisition of coordinated gestures and vocalizations at 12 months compared to HR-no ASD, HR-LD and LR-no ASD. Included a HR- Language Delay comparison group	Infants 9 h-ASD 13 h-LD 28 h- no ASD 30 LR no ASD	At 36 months met DSM-IV criteria, cutoff scores on ADOS and clinical best estimate	MacArthur-Bates Communicative Development Inventory (CDI) and MSEL administered. LR group recruited using different method (published birth announcement) compared to HR group	High (11)
Paterson et al., 2019	Between 6 and 12 months, HR-ASD engaged in less surgency behaviors (approach, activity level, vocal reactivity, smiling, laughter, pleasure and sensitivity) and more negative affect (sadness, distress) and less regulatory capacity (duration of orienting, soothability and cuddliness)	396 Infants 61 h-ASD 221 h- no ASD 114 LR- no ASD	At 24 months cutoff scores from ADI-R, ADOS and best clinical judgement were used to determine ASD diagnoses	Infant Behavior Questionnaire-Revised (IBQ-R) and AOSI, MSEL used to assess developmental and temperament measures respectively	Low (7)
Rowberry et al., 2015	At 12 months HR-ASD showed delayed imitation (motor, vocal and social imitation) compared to HR-no ASD All 3 h groups differed from LR on social orienting and receptive communication Parents of HR-ASD rate child’s social and communicative behavior atypical at 12 months	96 Infants 16 h-ASD 36 h-ATYP 19 h-TD 25 LR-TD	At 24 and 36 months cutoff scores on ADOS-G or ADOS-T	Infants recruited from ongoing prospective longitudinal study MSEL to assess overall development The First Year Inventory to assess overall development	High (10)
Sacrey et al., 2015	Parents of HR-ASD Reported greater atypical motor skills at 6 months and atypical sensory and play skills at 9 months differences in social concerns at 12 months, communication concerns at 15 months and repetitive behaviors at 18 months	237 Infants 62 h-ASD 106 h-no ASD 69 LR	At 36 months diagnosis conducted using DSM-IV-TR by clinician with 10 years of experience	Infants part of larger Canadian longitudinal study on early behavioral markers of ASD MSEL – ELC to assess overall development Vineland Adaptive Behavior Scales (VABS/ABC) to assess adaptive behavior	High (10)
Sacrey et al. 2020	At 9 months of age HR-ASD were less responsive to their name, engaged in less imitation, back and fourth vocalizations and eye contact than HR-no ASD and LR- no ASD at 9 months. Additionally, total APSI scores at 9 months predicted ASD outcome at 36 months with 70% accuracy. Parent concerns identified irregular play, language repetitive motor and sleep routine differentiated HR-ASD from HR-no ASD and LR at 9 months	136 Infants 31 h-ASD 51 h- no ASD 54 LR – no ASD	At 36 months diagnosis conducted using DSM-IV-TR by clinician with 10 years of experience	The Autism Parent Screen for Infants (APSI), The Parent Conerns Forms, ADOS & ADI-R were used to screen at 9 months	Mod (9)
Samango-Sprouse et al. 2015	Head tilt reflex (HTR) at 9 months was more indicative of ASD diagnosis at 24 months 60% of later diagnosed with ASD failed HTR at 9 months Accelerated Head circumference did not differentiate ASD from DD	1024 Infants 282 lost to follow up 14 ASD 33 DD 695 TD	At 36 months diagnosed by two specialists according to DSM criteria	Recruited through 3 large pediatric practices This study uses a LR population MSEL, PLS-4 and ELM-2 to assess neurodevelopmental function Infant/Toddler Sensory Profile and MacArthur-Bates Communication Inventory to asses parental concerns	Mod (9)
Sanefuji & Yamamoto 2014	At 13 months HR-ASD showed less imitation of body movement than TD group	39 Infants 21 h-ASD 18 TD	At 24 months obtained scores over the cutoff for ASD on the ADOS	Recruited through flyers, posters and local pediatric clinics Sample does not include a HR-no ASD group	Mod (8)
Wolff et al. 2014	At 12 months HR-ASD engaged in higher rates of repetitive behavior overall including self-injurious, ritualistic, restricted, and stereotypical RRB’s present across cognitive abilities and not negatively correlated with lower cognitive function at 12 months	250 Infants HR-ASD 149 h-no ASD 60 LR	At 36 months met DSM criteria and obtained scores over the cutoff for ASD on the ADOS	Infants part of ongoing Infant Brain Imaging Study RBS-R to assess repetitive behavior MSEL to assess overall functioning VABS-2 to assess adaptive functioning	High (10)

The participant classifications are as follows: *HR* high-risk by genetic disposition, *LR* low risk or no known delay or genetic disposition, *ASD* confirmed diagnosis of autism was established, no *ASD* a diagnosis was conducted but results were negative, *ATYP* development is not following a typical trajectory however no autism diagnosis was confirmed, *TD* typically developing and no known genetic disposition, *BAP* broadband autism phenotype, *DD* developmental delay with no autism, *LD* language delay with no autism

Inter-coding agreement for both inclusion and quality assessment were calculated by having each criterion assigned a plus if all coders agreed or a minus if they disagreed and then by dividing the number of agreements by the total number of agreed and disagreed criteria and multiplying by 100.

There was 100% agreement between coders for questions 1 to 5, which were single-item questions. For the quality assessment, all 26 articles were scored across 12 items, therefore resulting in a possible 312 items for agreement between coders. There was a 92% agreement between coders on all items and any disagreements were discussed among coders and a consensus was reached.

### Quality Assessment

As the nature of this research is cross-sectional and not experimental, a quality assessment tool developed by the National Heart, Lung and Blood Institute (NHLBI) and Research Triangle Institute for Observational Cohort and Cross-Sectional Studies based on other tools developed by researchers in the Agency for Healthcare Research and Quality (AHRQ) Evidence-Based Practise Centers, the Cochrane Collaboration, the United States Preventive Service Task Force, the Scottish Intercollegiate Guidelines Network, and the National Health Service Centre for Reviews and Dissemination (NHLBI 2017) was used with minor adaptations. The original 14-item quality assessment tool (NHLBI 2017) was modified by removing 2 questions that did not apply to the current research, therefore articles were given a score out of 12 based on the number of quality indicators which were present in the individual studies. The questions deemed non-applicable were question 8 and 9 of the original assessment tool. An article with a score of 10 to 12 would be assigned a “high quality” rating, a score of 7–9 would classify the study of “moderate quality” and a score of 6 or below would indicate a study of “low quality”.

### Breadth of Publications and Authors

The 26 selected studies for inclusion were published in 14 different peer-reviewed journals, with the most common journal being the Journal of Autism and Developmental Disorders in which 9 of the 26 articles had been published. Four journals; Infant Behavior and Development, The Journal of Child and Adolescent Psychiatry, Journal of Neurodevelopment Disorders and Autism, contributed with two publications each. The remaining ten journals hosted one article each: Molecular Autism, Journal of American Academy of Psychiatry, Journal of Abnormal Child Psychology, Autism Research, Psychology, Journal of Pediatrics, International Journal of Language and Communication Disorders, Journal of American Academy of Neurology and the Journal of Child Psychology and Psychiatry. Of the 26 articles, two articles had repeat first authors, Gangi et al. (2014) & Gangi, Schwichtenberg et al. (2018) and Sacrey (2015,2020).

## Results

The frequency and severity of autism symptoms in the HR-No ASD group often falls in the middle between the LR group and the HR-ASD group. The purpose of the current study is to highlight what ranges of symptoms differentiate the HR-ASD group from all other comparison groups, mainly the HR-No ASD and LR groups. Therefore, results will focus predominantly on the significant findings that were indicative of the HR-ASD group and will only briefly report symptoms found across the entire HR group.

The 26 included studies have been categorized into three domains on the basis of the symptoms identified in each study: social communication (12 studies), motor behaviors (7 studies), and parental reports (8 studies) with Ozonoff et al. (2018) appearing in both the social communication and parental report domains, as there were two unique components to this study. Within each domain, symptoms are presented by chronological age at which they appeared.

### Social Communication

Persistent deficits in social communication is the first stated diagnostic criterion for ASD (DSM-5; American Psychiatric Association 2013). Social communication encompasses, among other, shared affect and emotions, initiating and responding to joint attention, eye gaze, and facial expressions directed to others, with these skills emerging before vocal verbal behavior. At 7 months of age, infants pertaining in the HR-ASD group performed below comparison groups (HR-No ASD and LR) on two specific presses from the Autism Observation Scale for Infants (AOSI): an eye tracking task where the child has to track an object along a horizontal plane to the side and across midline and social referencing during the semi-structured assessment (Gammer et al., 2015). A similar outcome was found by Gangi, Ibañez and Messinger (2014) who reported that the HR-ASD infants between 8–12 months showed greater deficits in initiating joint attention without smiling and anticipatory smiling, reaffirming the difficulty that HR-ASD infants present in coordinating affect and gaze towards the end of the first year of life. Gangi et al. (2018) further explored gaze towards face across various contexts and reported that HR-no ASD and LR groups demonstrated increased social gaze with parent compared to an unfamiliar examiner; however, HR-ASD showed similar and lower levels of gaze across people (i.e., an unfamiliar examiner and parent) at 12 months of age, suggesting a difficulty responding to social context by not differentiating the frequency of social attention given to a familiar versus unfamiliar person. Additionally, infants who struggled to orient to their name being called at nine months of age were more likely to receive an autism diagnosis by 24 months and show greater impairment by 36 months of age compared to HR-no ASD groups (Miller et al. 2017). Furthermore, Filliter et al. (2015) provided additional support that HR-ASD infants at 12 months showed lower rates of smiling and positive affect compared to the HR-no ASD and LR groups and by 14 months of age HR-ASD showed marginally less engagement of attention and orientation to name than HR-no ASD during specific AOSI presses (Gammer et al. 2015). However, when examining HR-ASD behavior markers, more specifically total AOSI score, disengagement of attention and gaze following at 12 months, Bedford et al. (2016) reported these markers to reliably predict autism at 36 months in boys only but not in girls.

Between 13 and 15 months, HR-ASD infants showed greater delays in using gestures with 50% of the group failing to use any gestures and overall using less social interaction gestures and almost no joint attention gestures compared with LR and HR-no ASD group, showing a negative correlation between the frequency and type of gestures used at 15 months to severity of autism diagnosis at 24 months (Gordon & Watson 2015). Parladé & Iverson (2015) also found that between 12 and 14 months of age HR-ASD show significantly slower growth in coordinated communication such as combining eye gaze, facial expression, gesture and vocalization into a single communicative signal compared to HR-no ASD and LR-no ASD groups. Other research has also found similar difficulties with HR-ASD infants coordinating joint attention and vocalisations at 14 and 18 months of age which seem to be unique to HR-ASD compared with HR-no ASD or HR-LD (Heymann et al. 2018). Heymann et al. (2018) also noted that HR-ASD infants produced less frequent vocalizations and less complex vocalizations (primarily vowel-only vocalisations) at 14 months compared to their peers. At 15 months, lower rates of eye contact and lower positive affect continue to differentiate the HR-ASD group from HR-no ASD group (Nichols et al. 2014). Examining receptive and expressive communication at 12 months of age, Lazenby et al. (2016) reported that the HR-ASD group obtained significantly lower expressive and receptive scores on the Mullen Scales of Early Learning compared to the HR-no ASD and LR groups; however, there were several words that were more often understood or produced by the HR-ASD group compared to the other groups, although overall the HR-ASD group understood a significantly lower count of words.

In 2018, Ozonoff et al. prospectively examined onset patterns of ASD, more specifically regression patterns of social communication skills, and found that a loss of social communication skills such as shared affect and social engagement after 12 months of age was observed by clinicians in 88% of the HR-ASD sample whereas the HR-no ASD and LR groups did not show any regression in social-communication skills.

### Motor Behavior

When examining fine and gross motor behavior at 6 months of age, both HR-ASD and HR-no ASD showed motor delays in stationary tasks such as “pull to sit” and object manipulation tasks such as grasping compared to LR control group; however, the HR-ASD group also differed from both the HR-No ASD and LR groups in visual-motor integration tasks such as goal directed reaching (Lebarton & Landa 2019) Estes et al. (2015). also found deficits in gross motor skills to be the earliest indicative symptoms of autism differentiating the HR-ASD group from the LR-no ASD group by 6 months and differentiating from the HR-no ASD group by 12 months of age.

Accelerated head circumference during the first year of life has been previously identified as a biomarker of future diagnosis of developmental delays or ASD but has not been effective at differentiating the two (Courchesne et al. 2003; Elder et al. 2008).

Interestingly, when examined with head tilt reflex, a clearer differentiation between developmental delays and ASD emerged (Samango-Sprouse et al. 2015). Beginning around 9 months of age, when titled sideways, an infant begins to attempt to orient their head to stay upright, known as the head tilt reflex. It was estimated that 60% of the ASD sample compared to only 6% of the developmental delays sample failed the head tilt reflex test at 9 months (Samango-Sprouse et al. 2015). Using the Mullen Scales of Early Learning fine motor domain, a slower growth and reduced acquisition trajectory of fine motor skills was observed beginning at 14 months of age until 24 months in the HR-ASD group compared with other groups (Choi et al. 2018). Interestingly, Lebarton and Landa (2019), Samango-Sprouse et al. (2015) and Choi et al. (2018) all reported that increased fine and gross motor skills at 6 and 9 months were predictive of stronger expressive and receptive language at 24 months, suggesting that very early motor skills have a cascading effect for developing future language skills.

#### Restricted and Repetitive (stereotyped) Behaviors

Often called stereotypic behaviors, repetitive behaviors and restricted interests constitute the second autism diagnostic criterion listed after social-communication (American Psychiatric Association 2013). In a study conducted by Elison et al. (2015), at 12 months of age both HR-ASD and HR-no ASD groups were found to engage in more frequent stereotypic object manipulation behaviors compared to the LR group; however, the HR-ASD engaged in significantly more motor stereotypy than both of the comparison groups, indicating that motor stereotypy had greater predictive potential of an ASD diagnosis than object stereotypy. These results were further supported by Wolff et al. (2014) who reported that repetitive behaviors and restricted interests observed at 12 months of age in the HR-ASD group significantly differed from the HR-no ASD and LR groups in terms of frequency and topography, with the HR-ASD group presenting more stereotypical, self-injurious, ritualistic and restricted topographies.

#### Imitation

Imitation has been considered a core deficit in children with autism (Rogers et al. 2005) with the imitation of meaningless movements emitted by others (e.g. clapping or waving) being more difficult than imitation of movements involving objects (e.g. a wooden bird that produces a chirping sound when shaking up and down) (Rogers & Williams 2006). Similar findings have been observed in a HR-ASD group compared to a LR group, with the HR-ASD group at 13 months being more likely to imitate object play than the behavior of others, showing less overall imitation and following a delayed trajectory rather than an atypical trajectory. The types of imitation observed were consistent across both groups (HR-ASD & LR) showing that imitation which involved object manipulation was produced far more frequently than imitation involving meaningless movement at 13 months of age. (Sanefuji & Yamamoto 2014).

### Parental Concerns

Beginning from 6 months of age, parents of HR-ASD infants report higher rates of developmental concerns than HR-no ASD and LR groups (Sacrey et al. 2015). All three groups of parents reported similar types of concerns around similar ages, with sleep and motor concerns being reported in the first year of life and communication and challenging behavior concerns being more commonly reported in the second year of life. However, it was the number of concerns which separated HR-ASD from other groups. Parents of the HR-ASD group reported a significantly greater number of sensory and motor concerns at 6 months, sensory and play concerns at 9 months, sleep, sensory, social and play concerns at 12 months, sensory, communication and social concerns at 15 months, and sensory, motor, repetitive behaviors, communication, social, play and challenging behavior concerns by 18 months (Sacrey et al 2015). Notably, sensory concerns were the only domain that consistently differentiated the HR-ASD group from the others across all reported time periods. In addition to identifying social communication deficits as parental concerns at 12 months of age, Rowberry et al. (2015) reported that the biggest differentiation between HR-ASD and HR-ATP, HR-TD and LR-TD were deficits in overall imitation, including vocal, motor, facial and object imitation.

At nine months of age, the Autism Parent Screen for Infants (APSI, Bryson et al. 2006) and The Parent Concerns Forms (Sacrey et al. 2015) were able to predict ASD outcomes at 36 months of age with 70% accuracy (Sacrey et al. 2020). Four out of 26 questions on the APSI were found to distinguish HR-ASD infants from HR- no ASD and LR risk groups by 9 months of age, these questions examined responding to name, imitation, back and forth vocalisations and eye contact (Sacrey et al. 2020).

Del Rosario et al. (2014) and Paterson et al. (2019) examined temperamental trajectories of infants using prospective parental reports. Del Rosario et al. (2014) found an unusual and surprising temperament pattern distinguishing HR-ASD from HR-no ASD in that between 6–12 months the HR-ASD group was reported to be less active, more adaptable and more likely to approach socially unfamiliar targets than the HR-no ASD group. However, the HR-ASD group demonstrated a decreasing trajectory with regards to adaptability and approachability while the HR-no ASD group showed an increasing trajectory with regards to these temperamental behaviors (Del Rosario et al. 2014). Paterson et al. (2019) reported that beginning at six months and up until 24 months, HR-ASD infants exhibited lower surgency (approaching, vocal reactivity, smiling, laughter and high intensity pleasure) with the peak differences between HR-no ASD group at 12 months of age. Between 6 and 24 months, HR-ASD infants also demonstrated greater negative affect, including sadness, fear, and emotional reactivity (Paterson et al. 2019). Lastly, Paterson et al. (2019) found that HR-ASD infants at 6 months of age show reduced levels of regulatory capacity which includes but not limited to orienting to adult, soothibility and cuddliness (Table 2).

**Table 2:** Coding of included studies

Study	Q. 1	Q. 2	Q. 3	Q. 4	Q.5	Q.6
Bedford et al 2016	+	+	+	+	+	+
Bresnahan et al. 2015	+	+	+	+	+	+
Choi et al. 2018	+	+	+	+	+	+
Del Rosario et al. 2014	+	+	+	+	+	+
Elison et al. 2014	+	+	+	+	+	+
Estes et al. 2015	+	+	+	+	+	+
Filliter et al. 2015	+	+	+	+	+	+
Gammer et al. 2015	+	+	+	+	+	+
Gangi, Ibañez et al. 2014	+	+	+	+	+	+
Gangi, Schwichte et al. 2018	+	+	+	+	+	+
Gordon & Watson 2015	+	+	+	+	+	+
Heymann, et al. 2018	+	+	+	+	+	+
Lazenby et al. 2016	+	+	+	+	+	+
LeBarton & Landa 2019	+	+	+	+	+	+
MacDonald et al. 2020	+	+	+	+	+	+
Miller et al. 2017	+	+	+	+	+	+
Nichols et al. 2014	+	+	+	+	+	+
Ozonoff et al. 2018	+	+	+	+	+	+
Parladé & Iverson 2015	+	+	+	+	+	+
Paterson et al. 2019	+	+	+	+	+	+
Rowberry et al. 2015	+	+	+	+	+	+
Sacrey et al. 2015	+	+	+	+	+	+
Sacrey et al. 2020	+	+	+	+	+	+
Samango-Sprouse et al. 2015	+	+	+	+	+	+
Sanefuji & Yamamoto 2014	+	+	+	+	+	+
Wolff et al. 2014	+	+	+	+	+	+

In addition to studying regression, Ozonoff et al. (2018) examined the reliability of parental reports in general, concluding that these are more accurate when collected prospectively on current performance, using dimensional ratings (e.g. rating scale from 1 to 5 to reflect frequency; “when I call my child’s name, they look at me right away”) and less accurate when collected retrospectively and using a categorical rating system (e.g. since your last visit, has your child shown significant decreases in XX). When first year social communication skills such as social engagement were tracked prospectively using dimensional ratings, regressive onset comprised almost 90% of the HR-ASD sample; however, when asked retroactively and categorically if skills had been lost or regression occurred, only 30% of parents accurately identified that this had happened. Rather than solely incorporating developmental milestones at clinical checkups or retroactive questionnaires, assessing the same first year skills across multiple visits using dimensional ratings may provide significant insight into a loss of skills, signally a high probability of an ASD diagnosis. More specifically, when parents were asked prospectively to use a dimensional scale to report on their child’s behaviors, 69% of the HR-ASD parents’ responses were consistent with regression, however, only 46% of the same sample reported prospectively to categorical measures that their child lost skills. Most notably, when parents were asked retrospectively if their child had regressed in skills, only 29% reported regression (Table 3).

**Table 3:** Quality assessment scoring

Quality Assessment Items	1	2	3	4	5	6	7	8	9	10	11	12	Total
Bedford et al. 2016	1	1	1	1	1	1	1	1	1	0	0	0	9
Bresnahan et al. 2015	1	1	1	1	1	1	1	1	1	0	0	1	10
Choi et al. 2018	1	1	1	1	1	1	1	1	1	0	1	1	11
Del Rosario et al. 2014	1	1	1	0	0	1	1	1	1	1	0	1	9
Elison et al. 2014	1	1	1	1	0	1	0	0	1	1	1	1	9
Estes et al. 2015	1	1	0	1	0	1	1	1	1	1	1	1	10
Filliter et al. 2015	1	1	1	1	0	1	1	1	1	0	0	1	9
Gammer et al. 2015	1	1	1	1	0	1	1	1	1	0	1	0	9
Gangi, Ibañez et al. 2014	1	1	1	0	1	1	1	1	1	1	0	0	9
Gangi, Schwichte et al. 2018	1	1	1	1	1	1	1	1	1	1	0	0	10
Gordon & Watson 2015	1	1	1	1	1	1	1	0	1	0	0	0	8
Heymann et al. 2018	1	1	1	1	1	1	1	1	1	1	1	0	11
Lazenby et al. 2016	1	1	1	1	1	1	1	1	0	0	0	1	9
LeBarton & Landa 2019	1	1	1	0	1	1	1	1	1	0	1	1	10
MacDonald et al. 2020	1	1	1	0	0	1	1	1	1	0	1	1	9
Miller et al. 2017	1	1	0	1	1	1	1	1	1	1	1	1	11
Nichols et al. 2014	1	1	1	1	1	1	1	0	1	1	0	0	9
Ozonoff et al. 2018	1	1	1	0	1	1	1	1	1	0	1	0	9
Parladé & Iverson 2015	1	1	1	0	1	1	1	1	1	1	1	1	11
Paterson et al. 2019	1	1	0	1	0	1	1	0	1	1	0	0	7
Rowberry et al. 2015	1	1	1	1	1	1	1	0	1	0	1	1	10
Sacrey et al. 2015	1	1	0	1	1	1	1	1	1	1	1	0	10
Sacrey et al. 2020	1	1	0	1	1	1	1	0	1	1	1	0	9
Samngo-Sprouse et al. 2015	1	1	1	1	0	1	1	0	1	1	0	0	8
Sanefuji & Yamamoto 2014	1	1	1	0	0	1	1	1	0	0	1	1	8
Wolff et al. 2014	1	1	1	1	1	1	1	0	1	0	1	1	10

*Note.* Questions were: (1) Was the research question or objective in this paper clearly stated? (2) Was the study population clearly specified and defined? (3) Was the participation rate of eligible persons at least 50%? (4) Were all the subjects selected or recruited from the same or similar populations? Were inclusion and exclusion criteria for being in the study prespecified and applied uniformly to all participants? (5) Was a sample size justification, power description, or variance and effect estimates provided? (As observational cohort studies often are exploratory in nature, they often tend not to report about power or sample sizes, however this would still result in a “no” for this item) (6) For the analyses in this paper, were the *symptoms(s)* of interest measured prior to the outcome(s) being measured? (The symptoms were tracked prospectively and the outcome of ASD was measured at 36 months) (7) Was the timeframe sufficient so that one could reasonably expect to see an association between *symptom* and outcome if it existed? (i.e. observations spanned over months rather than days) (8) Was the symptom(s) assessed more than once over time before 18 months of age? (9) Were the outcome measures (dependent variables) clearly defined, valid, reliable, and implemented consistently across all study participants? Included an acceptable rate of intercoder reliability? (10) Were the outcome assessors blinded to the exposure status of participants? (Were the assessors blind to “High Risk” group membership?) (11) Was loss to follow-up after baseline 20% or less? (12) Were key potential confounding variables measured and adjusted statistically for their impact on the relationship between exposure(s) and outcome(s)? Omitted questions were: (1) For exposures that can vary in amount or level, did the study examine different levels of the exposure as related to the outcome? (e.g. categories of exposure, or exposure measured as continuous variable) (2) Were the exposure measures (independent variables) clearly defined, valid, reliable and implemented consistently across all study participants? (NHLBI 2017)

Gastrointestinal symptoms are reported more frequently in children with ASD compared to children with developmental delays other than autism or typically developing (Bresnahan et al. 2015; Ibrahim, Voigt et al. 2009; Wang et al. 2011). In a prospective maternal report, children between 6–18 months who would later be diagnosed with ASD were significantly more likely to experience at least one gastrointestinal symptom compared to children with developmental delays or typically developing (Bresnahan et al. 2015). Although gastrointestinal symptoms may often be categorized as a biomarker rather than a behavioral marker, some of these symptoms in children with ASD, such as constipation and disordered feeding patterns, are believed to, in part, have a behavioral etiology, i.e. gastrointestinal symptoms are stemming from or exacerbated by food selectivity, reduced fiber intake and atypical toileting practises rather than physiological differences in the gut (Buie et al. 2010; McElhanon et al. 2014; Wasilewska & Klukowski 2015). McDonald et al. (2020) further divided high risk membership to include single-incident families (only one older sibling with a confirmed diagnosis) and multiplex families (two or more siblings with a confirmed diagnosis). In the single-instance HR-ASD group, cognitive abilities as measured by MSEL scores and adaptive abilities as measured by VABS-II differed from single incident HR-no ASD by 12 months of age. The most significant findings of McDonald et al. (2020) were regarding prevalence of diagnosis, HR infants with two or more siblings were more than twice as likely to receive a positive diagnosis compared to single incident high risk infants. More specifically, only 33% of HR infants from multiplex families were considered typically developing by 36 months of age, highlighting the need for increased monitoring of infants with more than one sibling with ASD (McDonald et al. 2020).

### Quality Assessment

A modified version of the Quality Assessment Tool (NIH National Heart, Lung and Blood Institute 2017) indicated that 15 out of 26 studies were categorized as moderate quality, while the remaining 11 scored within the high quality range. Questions five, eight and ten saw the most frequent deductions in points across the body of literature. As explanation for the latter, the majority of the included studies were comprised of a HR sample, which consequently may lead to the researchers having previous interactions with the older sibling and/or family, thereby making it difficult to have researchers blind to the risk status of each participant. Some studies reduced the potential for this bias by having various items double-scored when blind assessment was not possible.

## Discussion

The results of the present systematic review of the literature provide a comprehensive review of early autism symptoms, as these are observed prospectively in HR-ASD infants. The majority of early symptoms research continues to use HR samples due to the comparative ease of recruitment and higher prevalence of an ASD diagnosis among these infants in comparison to the general population. The present study reports on 26 studies published between January, 2014 and May 2020 and 88% (23 out of 26) used a HR sample to study the earliest symptoms of ASD. Prospective HR studies have laid foundational groundwork for developing early detection screeners, albeit more research is still needed with LR samples to determine to what extent HR-ASD early markers are generalizable to LR-ASD populations. As much of the early signs research guides the development of early detection assessments and screening tools, we might be failing to identify ASD in populations that do not have an increased genetic risk, as this population would not necessarily be included in studies employing HR samples that very often involve the sibling of an older child diagnosed with ASD. Also, only two of the 26 studies separated genders when analyzing results. It is hypothesized that ASD presents differently in girls, both during the initial onset and in full manifestation of the disorder. Future efforts should focus on whether early symptoms present equally across both genders or which symptoms are differentiated between males and females.

### Regression of Skills as its Own ‘Early Sign’

Developmental regression refers to a loss of acquired skills that is not explained by traumatic brain injury or by distressing events. Up until recently, regressive onset was believed to be a rare occurrence in children with autism, this was in part due to a lack of a consistent operational definition or a standardized measurement tool to capture its occurrence (Zhang et al. 2019; Zwaigenbaum 2019). Furthermore, regressive onset can be more difficult to capture by clinicians, as it has usually already occurred prior to the parent seeking out treatment (Zwaigenbaum 2019). A major contribution to the understanding of the under-estimated prevalence of regressive onset ASD and how to more accurately capture a decreasing trajectory of skills, Ozonoff et al. (2018) have provided a framework with significant practical applications which can easily be adopted by clinicians. When using a prospective dimensional approach to monitor regression, regression of first year social communicative skills was reported in 88% of the sample by clinicians, in other words, 88% of HR-ASD infants showed losses in first year skills whereas, regressive trajectories were not observed in the comparison groups of HR-no ASD or LR-no ASD, implying that observed regression in any group other than HR-ASD would be quite rare and unexpected. However, how regression is being monitored and the rating system used is essential to proper detection. When first year social communication skills such as social engagement were tracked prospectively using dimensional rating, regressive onset comprised almost 90% of the HR-ASD sample; however, when asked retroactively and categorically if skills had been lost or regression occurred, only 30% of parents accurately identified that this had taken place. Rather than solely incorporating developmental milestones at clinical checkups or retroactive questionnaires, assessing the same first year skills across multiple visits using dimensional ratings may provide significant insight into a loss of skills, signally a high probability of an ASD diagnosis. Such an approach would also allow parents to complete the assessment remotely, enabling rural or low-resourced populations to access a reliable yet low effort and low cost means of identifying ASD early on. A review by Ozonoff and Iosif (2019) reiterate the point that regressive onset is now believed to be the rule rather than the exception.

### Motor Skills

Fine and gross motor skills continue to be among the earliest observable signs and have demonstrated consistent correlations to future expressive and receptive language skills by 24 months of age, signaling that early motor skills have a cascading effect on future language (Choi et al. 2018; Lebarton & Landa 2019; Rowberry et al 2015; Sacrey et al 2015; Sanefuji & Yamamoto 2014). The importance of early intact fine and gross motor skills continues to be consistently documented; thus, it is increasingly important to develop stronger interdisciplinary approaches to pre-diagnostic intervention to include a larger emphasis on pediatric occupational and physical therapists that can support the development of these pivotal skills.

### Parental Concerns

Research covering prospective parental concerns within the HR-ASD group has demonstrated validity over the course of many studies (Del Rosario et al. 2014; Rowberry et al. 2015; Sacrey et al. 2015). Parents are the experts on their children and have more knowledge about the child’s everyday functioning (Ozonoff et al. 2018), therefore their reports can be key in identifying infants at risk. Parental report should continue to be an imperative component in early detection screeners in addition to clinical observation. Additionally, infants with two or more older siblings with ASD were two times more likely to be diagnosed compared to infants with only one older sibling, thus children from multiplex families should be monitored diligently for early symptoms (Macdonald et al. 2020).

### Excesses and Deficits

The earliest markers of ASD often appear as skill deficits or decreased frequency of age appropriate behaviors rather than excesses of atypical behavior, making it more difficult to identify symptoms without comparing progress to a typically developing group. In the current review, 16 of the 19 studies reported on markers which are considered to be behavior deficits, i.e. deficits in eye gaze, attention, fine motor skills, social smiling, positive affect, visual tracking, joint attention, gestures, receptive language, visual motor integration, imitation and reflexes, meaning that the child is engaging in all of these behaviors however at a decreased frequency or duration, with an atypical topography or at a delayed trajectory in comparison to their typically developing peers.

On the other hand, only three studies reported on behavior excesses which included rigid and repetitive behaviors and gastrointestinal symptoms. The understanding of behavioral excesses, and their infrequent onset as an early symptom is critical to further developing early detection tools which capture both behavioral excesses but more importantly behavioral deficits. The earliest signs more often present in the form of deficits or decreased frequency of typical behaviors, rather than the excess or addition of atypical behavioral symptom such as rigid repetitive behaviors. Thus, with the majority of early symptoms emerging as deficits, early-screening tools should focus on comparing the frequency of typical behaviors (e.g. such as initiating joint attention, using gestures, imitating, etc.) in those at risk to expected frequencies of typical development.

### Notable Findings for Studies which were Excluded from Review

Dozens of additional important studies have been published in the previous five years but were not included in the current SLR due to not meeting inclusion criteria. Many studies either did not report on final diagnostic outcomes or their findings were not significantly differentiated between HR-ASD and HR-no ASD. Nonetheless, there are notable results that can have important implications for future research.

A systematic review of parent-infant interaction in infants at risk of autism focused on the differences in parent-infant interaction styles to determine how atypical emergence of social communication in the infant effects the interactive behavior of the parent across HR-ASD, HR-no ASD and LR groups (Wan et al. 2019). Overall, parental interactions with HR infants demonstrated lowered interactive reciprocity with infants who displayed preverbal communication delays, deficits in gestures use and vocal-gesture coordination and limited variation in babbling, in that when infants displayed such behaviors some parents responded with decreasing reciprocity and social input towards the infant (Landa et al. 2007; Ozonoff et al. 2011; Yoder et al. 2009). A study by Chawarska et al. (2016) evaluated differences in social attention for female HR siblings to further investigate the finding that females are four time less likely to be diagnosed with ASD than males. This study utilized eye-tracking devices to observe orienting to social stimuli at 6, 9 and 12 month intervals and found that HR females showed greater social attention towards faces than HR males but also more social attention than LR males and females. Although the results did not look at final outcome of diagnosis, the finding that HR female infants are engaging in higher than normal rates of social referencing could be a significant barrier to early diagnosis for females as it is often a lack of social orienting and eye-contact that trigger parental concerns.

A handful of studies have examined differences in vocalizations and cries within the first year of life, finding that HR infants and more specifically HR-ASD infants will exhibit shorter cry units than LR-infants although phonation, overall duration and frequency did not significantly differ across groups (Uniwin et al. 2017). A similar study examined canonical vocalizations across infants at risk and revealed that infants in the LR group engaged in significantly more variation and frequency of well-formed consonant–vowel syllables than the HR infants, however HR-ASD and HR- no ASD did not show significant differences from each other (Garrido et al. 2017).

Numerous studies have examined delays or atypical motor development in HR infants, while motor imitation ability at 12 months has been positively correlated to expressive vocabulary by 18 months across HR and LR groups (Edmunds et al. 2017; Ingersoll et al. 2008; McDuffie & Yoder 2010). The ability to engage in responding to joint attention such as following an adult’s point or eye-gaze and imitation together were better predictors of expressive language across HR and LR groups. This is believed to be in part due to the motor skills required to imitate others plus responding to joint attention are both needed to develop a more sophisticated social communication repertoire including higher levels of expressive language (Edmunds et al. 2017). Leonard et al. (2015) showed that poorer early motor skills tested on the MSEL at 7 months of age predicted poorer expressive communication outcomes at 18 months of age. As children move from crawling to being able to walk, they are better equipped to engage in a higher frequency of sharing objects with others, as their hands are free, and they have the ability to more quickly access the adult compared to crawling. However, HR infants at 14 months of age displayed greater deficits in postural control and engaged in fewer posture changes, less sophisticated postures and more time sitting than LR infants, leading to less frequent object sharing with adults (Srinivasan & Bhat 2016). Furthermore, motor abilities required to engage in object exploration differed in HR and LR groups as early as at 6 months and continued to develop differently through the first year of life. Typically developing infants displayed greater ability to grasp a rigid ball at 6 months and more purposeful dropping of objects at 9 months compared to HR infants (Kaur et al. 2015; Libertus & Shepard 2014). Object exploration is believed to have a cascading effect on communication and cognition and HR infants were found to significantly differ from LR infants at 6 and 9 months of age with regards to visual and oral object exploration (Korteba et al. 2014). Infants at 10 months of age differed in their ability to demonstrate a reach to grasp of preferred object with HR infants showing a delayed impaired ability of the skills compared to the LR controls Ekberg et al. (2016). Although motor impairments are not considered to be a key diagnostic characteristic of ASD, motor impairments seem to be consistently one of the earliest observable signs which differentiate HR from LR infants and could potentially provide a valuable addition to the current diagnostic criteria.

Other early signs research has examined the onset of sensory differences by examining the behavioral response patters or HR and LR infants to sensory stimuli. Atypical sensory responses include hypo-responsivity, hyper-responsivity and sensory seeking behavior. Sensory seeking behavior is defined as the enhancement or prolonging of a non-social object or event such as mouthing objects, visually examining spinning objects, or intensely rubbing various surfaces (Ben-Sasson et al. 2009). Atypical sensory seeking behaviors during the first 18 months of life were seen to be the most common sensory responses among HR infants and HR infants displayed significantly more sensory seeking behaviors than LR infants at 18 months of age (Damiano-Goodwin et al. 2018). Infant development relies on infants to understand the connection between their actions and the consequences presented by their environment by using previously obtained information and applying that information to new contexts. An inability to apply previous knowledge to changing contingencies could have significant impacts on the infant’s ability to acquire new behaviors. Using two identical looking baby rattles, one which made sound upon shaking and one that was silent, typically developing infants would show extinction burst behavior when given the rattle that was silent after they had engaged with the rattle which made a noise, demonstrating a generalized expectation that rattles should make noise based on their previous knowledge. However, HR infants did not demonstrate this extinction burst behavior and began shaking the silent rattle with a much lower intensity thereby not transferring expectations of a previous environmental contingency (Northup et al. 2017).

## Limitations

It is important to examine the quality of studies that comprise a SLR or meta-analysis. The quality assessment for the present SLR revealed that six of the 19 studies or approximately 32% of the studies obtained a moderate quality score whereas the remaining 68% of the studies were considered high quality. The predecessor literature review examining early signs of ASD research (Zwaigenbaum et al. 2015) not only does not utilise a replicable review methodology but also does not contain a quality assessment of the studies included.

The second limitation of the present SLR is that only two of the 19 studies (approximately 10%) used a normative population sample and not a high-risk sample. There is a logistical and statistical tendency for researchers to use a high-risk sample of children when studying early signs of ASD, however this can create limitations with the generalizability of the results. Of the 17 studies included in the SLR which used a HR sample, HR-no ASD and LR-no ASD developed as distinct groups with the HR- no ASD group showing less symptoms than the HR- ASD group however less symptoms than the LR- no ASD group. As it is difficult to recruit a large LR-ASD group for a prospective infant study, the majority of the research has been conducted with high risk populations therefore it is unclear to what extent the findings generalize to a low risk population (Zwaigenbaum et al. 2015). As much of the early signs research guides our awareness and dictates the development of early detection assessments and screening tools, we may be failing to identify ASD in low risk populations (such as the first child population) that do not have an increased genetic risk. The earliest signs of ASD appear to take on a heterogenous trajectory where no single sign has been identified as a reliable indicator when examined in isolation and instead assessing each additional early symptom as posing a cumulative risk has proven reliable. Acknowledging that ASD cannot yet be determined by one symptom underscores the importance of using early screening tools which examine cumulative risk. It is imperative that clinicians and practitioners are using these tools along with clinical judgment and parental reports to guide ASD risk and developmental surveillance.

Lastly, search terms were limited in scope to “early signs” or “early detection” or “early symptoms” and “prospective” and “autism” or “Autism Spectrum Disorder” or “ASD”. Using wider search terms could have led to more relevant studies being retrieved. In light of the fact that we only identified one additional reference as relevant through manual searches, we trust that the number of potentially relevant but omitted studies is negligible.

## Future Directions

Due to a heterogeneous and complex onset of the earliest signs of autism, no single sign has been identified as a reliable indicator when examined in isolation. One very important finding of this study is the fact that regression seems to be exclusive to HR-ASD and does not overlap with HR-no ASD. Future development of regression tools which use prospective and dimensional data of current frequency of social behaviors show promise in identifying greater true positive and fewer false positive cases compared to other screening tools (i.e. increasing specificity). However, more research is needed in this direction.

Additional established early behavioral symptoms of ASD, such as deficits in fine and gross motor control, joint attention, babbling frequency and variety of vocalizations, motor and vocal imitation, orienting to name and decreased positive affect, social smiling, object exploration and use of gestures should be understood as posing a cumulative risk, i.e. the more symptoms the greater the risk. Cumulative risk thresholds have been established and tested across multiple early detection tools. Therefore, it is imperative for early childhood stakeholders (doctors, nurses, teachers, clinician, etc.) to rely on the use of established early screening tools such as the M-CHAT-R/F (Robins et al. 2014), the STAT (Stone et al. 2004), the POEMS (Feldman et al. 2012), the ITC (Wetherby & Prizant 1993), or semi-structured tools such as the AOSI (Bryson et al. 2008), ADOS (Gotham et al. 2006), ADI-R (Lord et al. 1994), in order to understand the full presentation of symptoms. These screeners and assessments should be used to guide decisions regarding at-risk status and follow-up assessments together with clinical judgment and parental reports.

Future directions will need to examine if regression is more likely in a HR-ASD sample compared to a LR-ASD sample, i.e. whether a loss of skills is a good indicator of ASD regardless of risk status or whether regression is more uniquely a symptom within the children who already have a sibling with ASD. Additionally, an important future direction to further address this finding would be to develop a screening tool which will use prospective and dimensional reporting to track regression of skills over the first year of life. Enabling parents with the tools to prospectively track their child’s development could allow for earlier identification, diagnosis and treatment.

Furthermore, having additional ways to identify atypical development allows parents and practitioners to intervene prior to the full onset of the diagnosis.

Other key findings of the present SLR corroborate previous findings that delayed motor skills, or atypical stereotyped or repetitive motor skills are indicators of ASD (Choi et al 2018; Elison et al. 2014; Lebarton & Landa 2019; Sacrey et al. 2015; Samango-Sprouse et al. 2015; Wolff et al. 2014). Additionally, social communicative behaviors such as the use of gestures, eye gaze, social smiling, anticipatory smiling, responding to name, and various forms of imitation continue to make up the bulk of the research regarding the earliest symptoms, however often times these behaviors are still present in the HR-ASD population but reported as less frequently occurring than in HR-no ASD and LR comparison groups (Bedford et al. 2016; Filliter et al. 2015; Gammer et al. 2015; Gangi et al. 2014; Gangi, Schwichtenberg et al.^2018^; Gordon & Watson 2015; Nichols et al. 2014; Rowberry et al. 2015; Sanefuji & Yamamoto 2014). The understanding that the majority of social communicative early symptoms appear as deficits or are observed less frequently contributes to the difficulty of early identification of ASD symptoms, as infants who will later go on to receive a diagnosis are engaging in social communicative behaviors but often less frequently than typically developing children, making it difficult to the untrained eye to identify them. This provides further support to the notion that prospective dimensional screening tools may allow for more accurate observation of a current frequency of social communicative behaviors which can be compared against norms of neuro-typical development. Many of the current early symptom screeners such as the most commonly used M-CHAT, require a retroactive and categorically forced choice rather than a prospective dimensional rating. i.e. “Does your child look you in the eye for more than 1 s? Yes or No”. The accuracy of early screening tools could potentially be improved by changing such question to “In the next ten minutes tally the number of times your child looks you in the eye” and then have that frequency compared to typical norms. Further research would be required to test such hypothesis.

## Electronic supplementary material

Below is the link to the electronic supplementary material.

Supplementary file1 (DOCX 18 kb)
