# Supplementary material for: The Emergence of Autism Symptoms Prior to 18 Months of Age: A Systematic Literature Review
Source: J Autism Dev Disord. 2020 Jul 30;51(3):973–93. doi: 10.1007/s10803-020-04618-w (PMC7954747; doi:10.1007/s10803-020-04618-w)
Supplement: Supplementary file 1 — Supplementary file1 (DOCX 18 kb) [file 10803_2020_4618_MOESM1_ESM.docx]

Notes to the Reviewer:

Thank you very much for your positive and constructive feedback. I have addressed the specific feedback in the chart below and have highlighted these changes in yellow in the manuscript.

| Feedback from Reviewers | Response and Actions |
| --- | --- |
|  |  |
| I suggest that the authors revise the manuscript in accordance with APA 7th ed.  Headings level: Headings in the method sections on Level 2 (e.g., Systematic Search Procedures and Inclusion and Exclusion Criteria etc.) should be in bold and adjusted to the left. Not in italics and centered. | This is completed. |
| New to APA 7 is that you should write only the first author + et al. in the text if there are three or more authors, even if it is the first time they are mentioned in the text. E.g., (MacDonald et al., 2014). | This is completed. |
| Reference list: Include all authors up to and including the 20 first authors. If there are 21 or more, you shoud list the first 19 then … and then the last author.              Use only this format for doi's: <https://doi.org/xxxxx> | This is completed . |
| The study is well described, however my main concern is with the limited time-frame of inclusion of studies. Even if you point to the Zwauigenbaum review, you also correctly point out that this is not a systematic Review. Therefor your study is the first systematic literature review on this issue. As the literature on this field is older than 5 years, it seems strange to limit the inclusion to this time-frame. The study should have included a larger number of studies, or describe the rationale for this limitation to a greater extent. | Thank you for this piece of feedback and a longer time frame was strongly considered. Here is our rationale for the current time frame:  The previous literature review, although not systematic, was conducted using a 22-person expert panel comprised of some of the top researchers in the field of early autism research. The previous review examined 419 references and selected 24 articles  focusing on clinical trials of developmental/behavioral interventions (ie, not medications or trials of other biomedical therapies) that included children aged <36 months. “A scoping approach, with some discretion of the multidisciplinary expert working group, was used instead to select articles of highest relevance. Members of the working group reviewed the articles and evaluated their methodologic quality…, assessment of evidence quality focused on study design (retrospective versus prospective), measurement (eg, use of validated measures for both risk factors and diagnostic outcomes), and whether diagnostic outcomes were measured blinded to risk factor status. The working group also took into consideration whether findings were replicated across independent laboratories. Panel recommendations were based on this evaluative framework. During the conference, the working group offered draft recommendations for discussion, modification, and ratification by all attendees. Electronic voting was used to express opinions and guide consensus building”  As that review covered 24 articles and the present review of the last 6 years includes a total of 26 articles and is approximately 13,000 words. By expanding the time frame beyond the previous 6 years would lead to excessive breadth at the expense of much needed depth to more fully understand the earliest manifestations of the onset of autism.  Added to the manuscript:  Although [the previous literature review] was not systematic, 419 articles were reviewed by an expert panel of 22 researchers who are highly specialized in the area of early autism symptoms which allowed for a wider scope of articles to be included with some discretion in order to include the most relevant articles (Zwaigenbaum et al., 2015). Six years has now passed since its publication and the area of early behavior symptoms has continued to spark interest, leading to dozens more publications on the topic. As the previous literature was extremely comprehensive and covered 24 articles, the current literature review focused on the most recent findings rather than including redundant findings which have already been covered extensively in its predecessor.  Pg 4-5 |
| Tables and figures should be called "note". | Added a “note” to bottom of Table 1.  However APA 7^th^ edition still requires the use of Figure and Table as headings.  <https://owl.purdue.edu/owl/research_and_citation/apa_style/apa_formatting_and_style_guide/apa_tables_and_figures.html>  I am not sure if I am misunderstanding this comment? |
| P. 6. The search is not up to date and more than a year old. The authors should search March 2019-to date and add the respective studies found in the new search to the result | This is complete.  Search has now included up until May 31, 2020. 7 New studies were added  These studies are highlighted in yellow on the tables, their findings are highlighted throughout the results and discussion section.  Methods section and Prism table are updated to include this addition. |
| The sentence "…the search terms were purposely limited to "autism" or "Autism Spectrum Disorder" or "ASD" in order to focus and emphasize literature which was reporting confirmed ASD diagnoses in line with the most current diagnostic criteria." Should be cut from the manuscript. I think the authors should just state that the search terms have limitations and leave it at that. | This has been cut from the manuscript, it now reads as:  Pg 26.  Lastly, search terms were limited in scope to “early signs” or “early detection” or “early symptoms” and “prospective” and “autism” or “Autism Spectrum Disorder” or “ASD”. Using wider search terms could have led to more relevant studies being retrieved. In light of the fact that we only identified one additional reference as relevant through manual searches, we trust that the number of potentially relevant but omitted studies is negligible. |
| P 18-19. Regarding the regression section. The authors should take a look at the following paper that came out in a special issue on regression in Neuroscience and biobehavioral reviews last year. Other papers from . Zhang, D., Bedogni, F., Boterberg, S., Camfield, C., Camfield, P., Charman, T., ... & Goin-Kochel, R. P. (2019). Towards a consensus on developmental regression. Neuroscience and Biobehavioral Reviews, 107, 3-5. the papers by Iosif and Ozonoff and Boterberg et al. in the same issue might be worth a look regarding regression which seems to be far more common than what was earlier thought (e.g. stated in 2011ish by Ozonoff in an editorial in JCPP). I think also the discussion part of the manuscript on regression could be updated a bit considering papers from the S.I. in neuroscience and biobehav rev. | Thank you for the feedback on this special edition in Neuroscience. We have added the following references: Zhang (2019), Zwaigenbaum (2019) and Ozonoff (2019).   Developmental regression refers to a loss of acquired skills that are not explained by traumatic brain injury or by distressing events. Up until recently, regressive onset was believed to be a rare occurrence in children with autism, this was in part due to a lack of a consistent operational definition or a standardized measurement tool to capture its occurance (Zhang et al., 2019; Zwaigenbaum, 2019).  Ozonoff and Iosif (2019) reiterate the point that regressive onset is now believed to be the rule rather than the exception. |
